# Young people with intellectual disability and the internet: Challenges and opportunities in qualitative research

**DOI:** 10.1177/17446295221095714

**Published:** 2022-05-05

**Authors:** Åsa Borgström

**Affiliations:** Department of Social Work, Faculty of Social Science, 111124 University of Gothenburg, Gothenburg, Sweden

**Keywords:** Intellectual disability, internet, methodological challenges, opportunities, qualitative research

## Abstract

Conducting qualitative research on young people with intellectual disability and the Internet poses methodological challenges as well as opportunities. Based on memos from a qualitative study, this article focuses on identified gaps related to the challenges of informed consent, access to Internet arenas and using stimulus materials. Opportunities, in terms of flexibility and relationships, are discussed and problematized. The discussion shows that researchers may need to move out of their comfort zone and try nonconventional methods of data collection. It is important to be creative and innovative but also to look after the rights and interests of participants. Furthermore, take a non-directive approach and assume young people with intellectual disability to be experts on their own lives. Finally, the power imbalance between a researcher and participant should be considered and the researcher should ask him-/herself which perspective he/she wishes to present or ‘whose side are we on?’

## Introduction

Intellectual disability and the Internet is a relatively novel but growing research area. Though most research on the topic comes from North America and Europe (Borgstrom et al., 2019; Caton and Chapman, 2016; Shpigelman, 2018), the context of studies is sometimes unclear. Participants may be geographically located in one country but use online resources from another (Caton et al., 2019). However, a common feature of many studies is that methodological challenges are barely discussed.

Nonetheless, there are several methodological limitations in the research field: people with a particular interest in social media are over-represented in research, and research projects tend to focus on experiences of using social media and therefore do not represent ‘real-life situations’ (Caton and Chapman, 2016: 135). There is a call for more research involving young people with intellectual disability, for example by observation or taking a netnographic approach (Borgstrom et al., 2019). Digital inclusion is a central theme, but while the body of research on this issue is growing, most studies are survey based or qualitative and small in scale (Chadwick, 2019). Hence, methodological and conceptual approaches need to be expanded. This means including support workers in research and investigating interactions between people with intellectual disability, their support workers and the Internet, and enabling them to share their narratives and experiences of Internet use in meaningful, inclusive and empowering ways (Seale and Chadwick, 2017).

Until now, methodological challenges seem to have been mainly discussed in collaborative research. By engaging in collaborative research projects, individuals with disabilities gain access to the research process and have the opportunity to reflect, define their own social issues and be agents for initiating social change (Coons and Watson, 2013; Kramer et al., 2011; Ollerton, 2012). However, there are critical voices that emphasize that involvement in this type of research produces limited change at different levels (García Iriarte et al., 2014). Others problematize the terms and level of participation in this research (Peuravaara, 2015). Several articles highlight and discuss methodological and ethical issues in these areas (Dorozenko et al., 2016; Iacono, 2006; Morgan et al., 2014; Ramcharan, 2006; Schwartz and Durkin, 2020). A number of ethical issues are raised with regard to participatory photo-based research (e.g. Photovoice) on the Internet, e.g. that participants have to understand the risks and benefits of posting pictures and other content on the Internet (Ralph and Prevett, 2014).

However, relatively few contributions have addressed challenges associated with research involving young people with intellectual disability and the Internet. The focus of this article is the challenges and opportunities presented by research on young people with intellectual disability and the Internet. In previous studies, methodological challenges related to qualitative interviews have been viewed as ‘sources of error’ (Høybråten Sigstad, 2014: 199) or quantitative limitations. When involving people with disability in research, it might be necessary to innovate the interview situation and use alternative strategies and methods. These innovations might, in other contexts, challenge traditional ideas of what is acceptable in research (Høybråten Sigstad, 2014). Nevertheless, research involving people with intellectual disability requires ethical awareness and a self-critical approach (Sauer, 2000).

The foundations of this article are the author’s own study, young people with intellectual disability, self-presentations and Internet-related support. Three challenges were particularly salient during data collection: (1) informed consent, (2) access to Internet arenas and (3) the use of stimulus materials. Based on these three key issues, the aim of this article is to discuss methodological challenges related to experiences from the study, previous research and methodological literature. In addition, a fourth theme relating to opportunities is discussed: (4) flexibility and relationships in qualitative research.

## Methodological challenges and opportunities

This article is based on a study that takes a qualitative approach and interpretative perspective inspired by Taylor et al. (2016). The study was born out of ethnography (Hammersley and Atkinson, 2007) and social work practice research, emphasizing the relationship and interaction between researchers, practitioners and service users (Helsinki statement on social work practice research, 2014). Another angle of approach was an interpretative phenomenological analysis (IPA; Smith et al., 2009; Smith and Osborn, 2015).

The data collection (interviews/focus groups) was conducted in easily accessible, quiet rooms in the participants’ schools (staff room, library and empty classrooms), mostly free from interruption (sometime a teacher would drop in but close the door again). The interviews always started with a couple of minutes of informal conversation, and the questions were short, simple and unambiguous (often adjusted and further simplified during the interview). The primary format used was open questions (qualitative interviewing) with complementary closed questions (yes/no and multiple choice; Perry, 2004). The length of the interviews was adjusted on an individual basis depending on the situation (individual interview, pair interview and focus group) and varied between 1 hour and 30 minutes (pilot pair interview) and 21 minutes (individual interview). The author’s own experience of school social work (Isaksson, 2016) in special programmes for pupils with intellectual disability facilitated communication during the interviews, e.g. awareness of the fact that the interview process might require additional time and patience, the importance of clearly identifying oneself, using one’s usual tone and volume of voice and using simple and clear language. It was important that participants were treated in an age-appropriate manner, understood what was being said and were offered help in a sensitive and respectful manner (Tassé et al., 2005).

The research process started with two reference groups. The purpose was to involve young people with intellectual disability and professionals in the research process. The first group consisted of six young people with intellectual disability (five young men and one young woman) in an upper secondary special programme school. The aim of this group was to serve as a ‘sounding board’ and forum to test potential issues. This group met three times in 2016. The second group consisted of professionals (teacher, special teaching assistant, school social worker) and was primarily a forum to test methodological issues related to the interviews. This group met twice in 2017/2018. The collection of data started with two pilot interviews (one individual and one pair interview) and a pilot meeting with a female focus group in an upper secondary special programme school. The main data collection consisted of individual and pair interviews (including the pilot pair interview) as well as gendered focus groups (female and male). The focus groups met four times and varied in size between two and four participants.

This article is based on memos from the reference groups, individual pilot interviews and the pilot meeting with the female focus group as well as the main data collection. Altogether, 17 young women and 9 young men (aged 16-21) participated in the study during 2017/2018. The participants primarily attended national programmes in upper secondary special programme schools in the west of Sweden. In a Swedish context, every pupil with an intellectual disability has the opportunity to attend a national programme. If they require a more personalized education, they have the right to attend an individual programme (Swedish National Agency for Education, 2018). The project was approved by the ethics committee for West Sweden. The study was adapted to comply with rules and guidelines of human subjects research from the Swedish Research Council, specifically requirements for information, consent, confidentiality and usage of data (CODEX rules and guidelines for research, 2021).

Methodologically, the study was characterized by an ‘adaptive theory approach’ (Layder, 1998: 47). Memo writing, meaning detailed commentaries of the meaning of data and their theoretical implications (Layder, 1998), has been central during the data collection. Memo writing is about making notes mainly for oneself, highlighting questions, problems and connections between concepts or categories in the data and codification process. In this way, discussion and self-dialogue creates a connection between theoretical reflection and practical issues during data collection and analysis (Layder, 1998). In this study, theoretical memos took the shape of personal notes made in a digital notebook (Word document). Notes were taken throughout the research process, from the reference groups to the pilot interview and the pilot focus group and main data collection. Theoretical memos may also include marginal notes made when analysing transcripts of documents (Layder, 1998). In this instance, marginal notes were taken during the transcription of the data, and every transcript (Word document) was annotated. The software NVivo 12 Pro was used to store and organize memos and marginal notes. Thus, a combination of methods (Layder, 1998) was used. To conclude, writing memos gives an overview of data and analysis and enables gaps in the data to be identified (Layder, 1998). In this study, these gaps were generally a result of challenges during the interviews and focus groups, including limitations in communication and difficultly sticking to a common thread, dominant participants accompanied by silent participants and/or participants repeating their answers. Other identified gaps were challenges concerning informed consent, access to Internet arenas and using stimulus material. The latter will be discussed in this section with regard to the following questions: What are the challenges? How should these issues be addressed? What are the considerations? What are the approaches? A concluding discussion will investigate the importance of flexibility and relationships in this research field.

### Challenges concerning informed consent

Challenges and risks increase exponentially in research that involves marginalized communities and populations (Pitts and Smith, 2007). With regard to young people with intellectual disability, informed consent is a potential issue. People with disabilities are often considered incapable of self-determination, including providing consent (or not) for research participation. Additionally, young people are often judged to be incapable of providing informed consent before the age of 15, 16 or 18 (Pitts and Smith, 2007). So, how should researchers deal with this dilemma? With regard to recruitment and consent, a number of measures are recommended, including the use of simple and easy-to-understand language, taking a multi-modal approach, recruiting at familiar community sites and involving researchers with experience of working with people with intellectual disability (Swaine et al., 2011). Gatekeepers, support persons and dual and group consent may also facilitate the process of obtaining informed consent (Carey and Griffiths, 2017), for example decisions on voluntary informed consent could be made by informants and familiar support persons together (Høybråten Sigstad, 2014). Moreover, a systematic and holistic approach can allow persons with intellectual disability to participate in the consent process to the best of their ability (Ho et al., 2018).

This study follows the rules and guidelines for human subjects research from the Swedish Research Council, specifically requirements for information and consent (CODEX rules and guidelines for research, 2021). The principle of informed consent ensures that peoples’ ability to make their own decisions is respected and that participants do not come to harm (Kvale and Brinkmann, 2014). The author obtained informed consent from all participants in the interviews/focus groups; the information was made available in accessible language; and the aim and method of the study and that participation was voluntary was made clear. Legal guardians of pupils under 18 years of age were informed of the details of the study by the relevant teachers (gatekeepers). A preliminary schedule and meeting place were agreed (Taylor et al., 2016). The participants were informed of the length of the interviews (20-45 minutes), that the interviews would be conducted at their school, of the motivation for and purpose of the study (Taylor et al., 2016) and that the results would be published in books and articles in Swedish and English. The concept of anonymity was also explained (Taylor et al., 2016) and that participants’ full names and the name of their school would not be published (pseudonyms were used for the participants in the study).

Understanding and completing the informed consent form was a challenge for the participants in general. One of the participants just wrote his first name, others did not remember their phone number and several did not know their email address (one did not write down the name of the school). Participants also found the design of the informed consent form concerning online observations difficult to understand. It seemed difficult for them to distinguish between questions about social media use and consent to observations on different social media platforms (e.g. Facebook and Instagram). Gathering correct usernames and personal links for Internet accounts was another issue. In some cases, participants gave consent to observations but did not specify usernames or personal links. In other cases, the specified names and links did not work. These issues highlight the additional time and patience that may be required when conducting an interview process with young people with intellectual disability (Tassé et al., 2005), as well as the importance of using language and interview design that are easy to understand (Perry, 2004; Swaine et al., 2011; Tassé et al., 2005).

Another challenge was to provide participants, both over and under 18 years of age, with written information (in an easy-to-read and extensive version) about the study. As an illustration, one participant (over 18) forgot the information on the table in the interview room; the author took the paper and tried to give it to the him in the corridor, but he refused to take it. According to the principle of informed consent, peoples’ ability to make decisions should be respected (Kvale and Brinkmann, 2014). For this study, every participant over 18 made his/her own decision about receiving oral and/or written information. In the case of participants under 18 years of age, teachers communicated information relating to the study to their legal guardians. With regard to the interviews and focus groups, the participants received oral and written information to take home. However, in the female focus group, none of the participants under 18 years of age wished to receive the written information. One of them (age 17) said that her parents had already received the information and another (age 17) stated that it was unnecessary because they were not very proficient in the Swedish language. Cases such as these raise ethical questions: should researchers contact and provide written information to legal guardians independently? If not, should a student be excluded from participating in the study? There is no single answer to these questions. In this case, the author strived to ensure that the participants did not come to harm (Kvale and Brinkmann, 2014) by weighing the pros (make their voice heard) and cons (not involving legal guardians). This illustrates the importance of innovation in this research area and of using alternative strategies and methods that might challenge traditional ideas of what is acceptable in research (Høybråten Sigstad, 2014). This is relevant when researching young people with intellectual disability, as well as other marginalized communities and populations (Pitts and Smith, 2007).

### Challenges of access to Internet arenas

In this relatively new area of research, there are few articles problematizing methodological and ethical issues. Of those that do exist, the majority focus on participatory image-based research, more specifically photovoice. Some researchers argue that photovoice may offer a powerful way of identifying and documenting the concerns of people with intellectual disability (and their families, carers and advocates). Internet publication can be supplementary to this (Boxall and Ralph, 2009). Posting images on the Internet involves both opportunities (cheap access to photography and web) and risks (e.g. changed and digitally manipulated images, cyberbullying and disablism). It is clear that photovoice may enable the perspectives of people with intellectual disability to be shared in research, but the methodological and ethical issues that it raises have to be carefully considered (Ralph and Prevett, 2014). It is important to emphasize the need for the international research community to be involved in ethical issues in this area as well as procedural approaches to research governance (Boxall and Ralph, 2009). With regard to young people with intellectual disability, qualitative research techniques may facilitate a deeper understanding of online behaviour, particularly a combination of participatory observation, deep hanging out, conversional interviewing and visual elicitation. However, these methods generate several ethical issues: the dilemma between privacy and well-being and completing the study and the informal rather than professional relationship between respondents and researchers (De Groot et al., 2019). This is reflected in a study about researcher and participant relationships on social networking sites, which highlights the importance of responding to literature and having a dialogue with participants on ethics and methodology to improve research design ‘on the run’ (Robards, 2013: 231). Ethical considerations in offline research may be transferable to online research, but this would have to be informed and reflective (Robards, 2013).

The intention of this study was to combine an ethnographic (Hammersley and Atkinson, 2007) and netnographic (Berg, 2015; Kozinets, 2020) approach. Netnography is a specific type of qualitative social media research that applies methods of ethnography and other qualitative research practices to the cultural experiences included and reflected within social media. This differs from general digital investigations in that it focuses on online traces and the Internet as an arena for social interaction (Kozinets, 2020). The rationale was that a combination of an ethnographic and netnographic approach might facilitate access to participants’ Internet arenas as well as provide opportunities to study the tension between online and offline activities. In the literature, there are four types of studies of online groups and collectives: firstly, studies of online interaction with modest participation, secondly, studies of online groups and collectives with certain participation, thirdly, studies of online groups and collectives with certain participation with additional interviews (online or offline), and lastly, studies of online groups and collectives along with offline research methods and additional interviews (online or offline; Bryman, 2016). This study is consistent with the latter and the purpose was to carry out online observations in public and semi-public environments, e.g. social networking sites (Sveningsson Elm, 2009). This study follows the rules and guidelines of human subjects research from the Swedish Research Council (CODEX rules and guidelines for research, 2021) and ethical requirements by the Association of Internet Researchers (AoIR) regarding respect for persons, beneficence and justice. Respect for persons grounds primary Human Subjects Protections: protecting peoples’ identities via anonymity, confidentiality and informed consent (Franzke et al., 2020). This association highlights that the greater the vulnerability of the community/author/participant involved, the greater the obligation of the researcher to protect the vulnerable group (Markham and Buchanan, 2012). Thus, taking a netnographic approach to a vulnerable population requires continuous ethical consideration, weighing benefits against risks (Kozinets, 2020). This was a guiding principle during the data collection.

Most of the participants in the study had accounts on social networking sites: Facebook, Instagram, Snapchat, YouTube and musical.ly and ask.fm. Some did gaming and vlogging (videoblogging) and blogging. During the research period (2015-2018), the digital use pattern among young people on social media changed from text messages to mainly pictures and videos. Several of the participants gave consent to online observations, but only 4 of 26 participants (including the pilot interview) provided functioning personal links and user names. These participants (young women) provided links and user names to public accounts, interview videos, music videos and make-up videos on YouTube. One participant uploaded vlogs on YouTube, and another had her own blog.

Interactions with the reference group indicated that it would not be easy to get access to the participants’ online arenas. In the reference group, the participants brought their mobile phones, but none wanted to show their social activities on the Internet, preferring to provide verbal descriptions. In the main data collection, consent regarding online observations was obtained in connection to the interviews, but this did not always mean access to Internet arenas. For instance, one participant consented to being friended/followed on Facebook/Instagram but not on Snapchat, where he interacted with his friends. Two other participants consented to observations on public accounts (on Instagram and YouTube) but not on Snapchat, because they considered it too private. Studies of online groups and collectives together with the use of offline research methods and additional interviews (online or offline; Bryman, 2016) generates new ethical and methodological challenges. One issue might be participants not admitting a researcher (a stranger) to their private online arenas; on the one hand this is sensible, as it is a question of personal integrity, on the other, it might pose a dilemma between privacy and well-being and completing the study (De Groot et al., 2019), in this case the online part. Another access issue was technical difficulties: one participant said she had no Wi-Fi in school, although today most schools in Sweden do (Swedish National Agency for Education, 2019). Another participant told the researcher that he had posted a ‘cat film’, which was searchable by his name, but the researcher never managed to find the film. These instances suggest that qualitative research, in this case online observations, may require additional time and patience (Tassé et al., 2005).

Another ethical and methodological issue was about transparency, friending/following participants, and online confidentiality. Firstly, becoming a friend or a follower with a person with intellectual disability online in research purpose requires a delicate balance. The participant might think that you are now actually friends. That is what it says in the online forum, but the abstract understanding of this is more difficult to comprehend. However, the intention was to be transparent with the research presence during online observations. But being transparent and becoming a friend or a follower with the participants at the same time as guaranteeing confidentiality was complicated. As an illustration, if a participant added the researcher on a social networking site, his/her participation in the study became relatively apparent to his/her other friends/followers. The question of confidentiality in combination with difficulties to do with consent led to a change of course and improvisation ‘on the run’ (Robards, 2013: 231). Instead of friending/following participants in semi-public environments (e.g. private accounts on Facebook/Instagram), the researcher focused on conducting observations in public environments (e.g. public channels on YouTube; Sveningsson Elm, 2009).

### Challenges using stimulus material

Focus groups are a common qualitative method in this research field, often combined with other qualitative methods (e.g. interviews). However, there are few studies based exclusively on focus groups with people with intellectual disability, and often a range of methods and participants are included, for instance semi-focus groups with people with intellectual disabilities and structured interviews with formal caregivers (Heitplatz et al., 2021); focus groups with students with learning disabilities, parents and staff and interviews with a student and staff member (Caton and Landman, 2021); focus groups with staff and pair and individual interviews with parents (Löfgren-Mårtenson et al., 2015; Molin et al., 2015); and focus groups and individual interviews with staff (Ramsten et al., 2019).

A recently conducted study about digital media use implemented focus groups in combination with the Talking Mats Method (Heitplatz et al., 2021; Talking Mats, 2013). Here, images (e.g. smartphones, tablet computers or logos from social media websites) with subtitles and symbols were used to support questions (in plain language) by the interviewer (Heitplatz et al., 2021). Another study, about Internet safety and online radicalisation, was mainly based on exploratory focus groups. These focus groups followed the same schedule and were implemented in two steps. The first explored Internet use: activities, where participants used the Internet, what devices participants used and perceptions of Internet safety. The second was a more detailed exploration of what participants knew about Internet safety: risks, grooming and online radicalisation and perceptions of the value of learning about these risks (Caton and Landman, 2021).

A mix of methods are proposed to highlight the voices of people with intellectual disability (Ottmann and Crosbie, 2013). The author’s study used a multi-method design (Bloor, 2001) that included a mix of qualitative methods: individual interviews, pair interviews, focus groups and online observations. Focus groups can be difficult to organize and are likely to produce uneven results (Ottmann and Crosbie, 2013). However, they are ideal for exploratory studies in new research areas (Kvale and Brinkmann, 2014). The focus groups in this study included young people with intellectual disability and a moderator (the author). The interview questions were in a relatively narrowly defined area (social activities on the Internet), and the focal point was interaction and common creation of meaning (Bryman, 2016). Usually, focus groups consist of six to eight participants with similar backgrounds (Morgan, 1998), but in this case they included between two and four participants and were gendered (all-male and all-female groups). All participants attended an upper secondary special programme school. Despite this, the groups were quite heterogeneous. The biggest differences affecting the group were the participants’ personalities (e.g. dominant, silent). Focus groups are characterized by a non-directive interview approach, in which the moderator (e.g. researcher) introduces topics for discussions (Kvale and Brinkmann, 2014; Morgan, 1998). In this study the topics were: self-presentation, relations on the Internet, opportunities and risks, and online and offline identity. A crucial ethical concern in focus group research is privacy, and it is important to guarantee participants’ confidentiality (Morgan, 1998). This study followed rules and guidelines of human subjects research from the Swedish Research Council, specifically requirements for confidentiality (CODEX rules and guidelines for research, 2021). Additionally, common rules were stated in easy-to-understand language at the first meeting of each focus group, for example ‘everything we discuss stays within the group’ and ‘you can share as much as you want’. In multi-method design, focus groups might be an alternative by which to deepen earlier analysis (Bloor, 2001). In this study, the intention was for the interviews, focus groups and online observations to be complementary.

The intention was to use stimulus material in the focus groups. Using stimulus material is an accepted method for use in focus groups with people with intellectual disability (Heitplatz et al., 2021). The chosen material was about young people with intellectual disability, participation and safety online and included five short films and a guide with proposed questions. The aim of the material was to enable discussions about online behaviour and allow participants to share experiences and strategies of online safety (Bureau Against Discrimination, 2021). The first film, ‘Keep in contact – Elementary about social media’, was used reasonably successfully. For example, the participants related their own mothers to the ‘mother character’. However, it was difficult to initiate an exchange of views and the questions ran out quickly. It is unclear why the stimulus material did not work in this study, but some issues are worth highlighting.

Firstly, the author’s experience of school social work (Isaksson, 2016) may have affected the discussions, although it may also have helped to facilitate the implementation of the interviews and focus groups, e.g. making sure that participants understood what was being said and offering help in a sensitive and respectful manner (Tassé et al., 2005). In this case, using pre-existing material seemed to affect the role of the researcher. Leading discussions from pre-existing films/questions is an exercise that was associated with the author’s former role as a school social worker (Isaksson, 2016; e.g. advising young people in online behaviour), and this jeopardized the independence of the role of researcher. Secondly, the female participants had difficulties identifying with the main character, i.e. a young man with intellectual disability*.* Recent research emphasizes a contradictory identification process among young people with intellectual disability, who use civil and non-institutional arenas to fulfil their aspirations of ‘being someone’ or belonging to something outside of an institutional setting (e.g. special school programme; Molin, 2020). The participants’ resistance to identifying with the main character could be understood as an unwillingness to identify with other young people with intellectual disability, or it could be an issue with identifying across genders. The fact that the main character was a young man might have made it more difficult for them to identify with the film.

However, a non-directive interview approach is significant for focus groups and implies a moderator (e.g. researcher) introducing topics for discussion (Kvale and Brinkmann, 2014; Morgan, 1998). In this case, it seemed that the stimulus material was too directive, which impeded discussion. Consequently, the approach to the focus groups was changed. Instead of proceeding from the stimulus material, the author decided to initiate the discussions from the topics in the interview guide (self-presentation, relations on the Internet, opportunities and risks, and online and offline identity) and by using open questions with complementary closed questions (yes/no and multiple choice; Perry, 2004). This clarified the role of the researcher and ensured a non-directive approach that started with the participants themselves, assuming them to be experts on their own lives.

## Flexibility and relationships

Recent research emphasizes the importance of flexibility and building relationships (Carey and Griffiths, 2017; Corby and Sweeney, 2019; Hall et al., 2017; Ho et al., 2018; Hollomotz, 2018; McDonald et al., 2013). One study suggests a flexible model for engaging persons with intellectual and developmental disabilities in research. Researchers should plan for variety, i.e. strive for varied recruitment strategies and means of presenting information, but also take time to plan and execute these approaches and consider the funding needed to provide resources (McDonald et al., 2013). Another study highlights the importance of flexible contact methods; e.g. digital channels such as emails and social media, as well as recruitment via charities, families, support groups and multi-site organizations. Moreover, it suggests building stronger relationships between care companies and universities to spread research findings and encourage participation in studies (Hall et al., 2017). The use of adapted communication strategies during the consent process seems to be important for building consensus and later engagement with participants (Ho et al., 2018). A flexible approach to interviews is proposed, including getting to know how each participant communicates best, respecting what he/she has to offer and having faith in the participant’s ability to mediate his/her own subjectivity (Hollomotz, 2018). One example of a flexible method is shadowing, which may facilitate recruitment, provide insight into daily life, give a voice to people with intellectual disability and break the traditional roles of researcher and participant (Van der Weele and Bredewold, 2021). Another study argues that despite knowledge of the best way to inform potential participants of the study and having people with suitable experience in the research team, it is important to be flexible over data collection methods and time of collection and to build relationships with staff and families (Corby and Sweeney, 2019). There is also a need to build effective relationships between the people with intellectual disability who participate in research and the services (service managers, gatekeepers and staff) that support them. Deep empathic communication with participants is central to generating meaningful data and may help to make the research process more enjoyable for participants. In addition, relationships with support services are important for enabling the research to go smoothly (Carey and Griffiths, 2017). Moreover, establishing confidence is central to trust and effective communication in an interview situation (Høybråten Sigstad, 2014).

Flexibility and building relationships were cross-cutting elements during the research process. When approaching the field, the author used varied recruitment strategies (McDonald et al., 2013) and flexible contact methods (Hall et al., 2017), e.g. personal contacts, email and phone calls, in-person presentations at staff meetings (and in staff rooms) and presentations to pupils in classrooms and in corridors. Regarding flexibility, the first example from the data collection was the interview design. Initially, the intention was to conduct individual interviews, but in the second pilot interview, the female participants surprised the author by asking to be interviewed together. The author was hesitant at first, but they insisted, saying that they already knew everything about each other and that there were no secrets. Once the interview had started, it became clear that it might be hard to follow the interview guide. It was obvious that the participants wanted to tell ‘their story’, and the author let them do that. In this way, the author embraced a flexible approach, getting to know how the participants communicated best, respecting what they had to offer and having faith in their ability to mediate their own subjectivity (Hollomotz, 2018). Telling ‘your story’ was a recurrent theme during the interviews. Yet, allowing the participants to tell their story was not an aim in itself. Instead, it seemed to be a desire from the participants. In another interview, it was hard to follow the interview guide because the participant started to talk about other topics (e.g. sexual abuse). In this case, the author had to clarify the role as a researcher as well as the agenda with the conversation and direct the interview to provide balance. Nevertheless, at the end of the interview (1 hour and 8 minutes), the participant wanted to continue. Previous research highlight that the closeness in the interview situation, which is useful regarding to information disclosed, might also be deceptive by arousing emotions like those in friendship (Barron, 1999). This poses questions of whether flexibility is always worth striving for, but also issues of power. In relation to marginalized participants, it is important to recognize ‘the researcher’s superior position of power’ (Barron, 1999: 48). However, this comes down to the question of whether there are any situations in which the researcher has to use power and direct the interviews (e.g. to protect the participants), and if it is possible to assert a position of power without dominating the perspectives presented. These issues need further investigation and require us to ask ‘whose side are we on?’ (Becker, 1967; Taylor et al., 2016: 26).

A starting point of the study was to combine an ethnographic (Hammersley and Atkinson, 2007) and netnographic (Berg, 2015; Kozinets, 2020) approach. Establishing rapport is the goal of every field researcher, and in most research fields this takes time. Accommodating participants’ routines and ways of doing things is a prerequisite. The easiest way to build a relationship is to establish what you have in common. Additionally, it is important to be humble and show interest (Taylor et al., 2016). In this study, effective relationships (Carey and Griffiths, 2017) were essential to gain access to the research field. In relation to the reference group and during data collection, the author invested in building relationships with head teachers and responsible teachers (gatekeepers). The author also had a reference group of professionals (teacher, special pedagogue and school social worker) and presented the project to the school health service (elevhälsoteam). Establishing and maintaining rapport is an ongoing activity for which there are tactics, for example asking naïve questions, putting yourself in situations likely to yield interesting data, doing favours to get access to information, not letting participants know the specific focus of your study and using aggressive field tactics (e.g. structured interviews) for a later stage of research (Taylor et al., 2016). In this study, these activities were not particularly salient. The data collection started with interviews (individual and pair interviews) and focus groups. The author met with most of the participants only once, except for the focus groups, which met four to five times each. The following example is from the third meeting with the male focus group. The author started the recorder and the participants opened up and even shared their negative online experiences. Afterwards the author met a teacher in the corridor and shared the positive experience. The teacher confirmed similar experiences and emphasised the positive feeling of student participation as well as the negative feelings when students are silent, saying ‘maybe they want to look at you for a while before they start to talk’. The scenario described above can serve as an illustration of the importance of establishing and maintaining rapport (Taylor et al., 2016), as well as establishing confidence to create trust and facilitate communication in the interview situation (Høybråten Sigstad, 2014).

## Conclusion

This article focuses on methodological challenges of qualitative research methods (interviews, pair interviews, focus groups and online observations) with and about young people with intellectual disability. Specific challenges, regarding informed consent, access to Internet arenas and using stimulus material, are discussed in relation to previous research and method literature. Additionally, opportunities in terms of flexibility and relationships are discussed and problematized.

The discussion in this article has demonstrated the challenges of understanding and completing informed consent as well as providing written information. It raises questions about the requirements of informed consent and whether it always has to be applied to the letter. Carrying out studies with and about young people with intellectual disability entails certain demands. As a researcher, you may need to step out of your comfort zone and try non-conventional methods. This applies not just to young people with intellectual disability but also to other marginalized groups. This issue needs to be higher on the social work research agenda.

Another challenge was gaining access to Internet arenas. Here, it is crucial to be creative and innovative while looking after the rights and interests of the participants. This places an emphasis on personal integrity. It is important to be transparent and have a distinct research presence, online as well as offline. Online, this raises questions about the requirement for confidentiality. The Internet is a novel research area, and conventional method literature seems insufficient to some degree. Thus, this is an area for further research.

Practical experience in the research area has both advantages and disadvantages. Primarily, it is important to clarify the role of the researcher, specifically in an investigative rather than consulting role. With regard to stimulus material, it is important to appeal to the heterogeneous group of young people with intellectual disability. Perhaps most important is to have a non-directive approach and start with the participants themselves, assuming young people with intellectual disability to be experts on their own lives.

Finally, flexibility and building relationships are the main prerequisites in this research area. However, sometimes the researcher has to be more or less directive (e.g. to protect the participants). In these situations, it is crucial to consider the power position of a researcher, requiring you to ask yourself which perspectives you want to present. This may be an area for further research and raises the question ‘whose side are we on?’ (Becker, 1967; Taylor et al., 2016). To sum up, there is an urgent need for continued research in this area, not only on problems related to young people with intellectual disability and the Internet but also on methodological and ethical issues.
